# Supplementary material for: The genome sequence of the Light Arches, Apamea lithoxylaea (Denis & Schiffermüller), 1775 (Lepidoptera: Noctuidae)
Source: Wellcome Open Res. 2025 Sep 15;10:508. [Version 1] doi: 10.12688/wellcomeopenres.24878.1 (PMC12784050; doi:10.12688/wellcomeopenres.24878.1)
Supplement: Peer review history

# Reviewer report: Reviewer response for version 1

**DOI:** 10.21956/wellcomeopenres.27403.r134566

**Published:** 2026-01-08

**Authors:** Annabel Whibley

**Affiliations:** 1 Grapevine Improvement, Bragato Research Institute, Lincoln, Canterbury, New Zealand

Boyes and colleagues report the genome assembly of *Apamea lithoxyaea *(the noctuid moth commonly known as Light Arches).  Two haplotypes are reported, with haplotype 1 scaffolded to pseudochromosome level and including both W and Z sex chromosomes since the individual sample was female. Sample identitfication was verified by barcoding and followed the standard DToL pipeline for sample processing, sequencing with the ultra-low-input PacBio Hifi protocol and HiC, and assembly.

 As seems often the case, the spectrophotometric ratios deviate from typical expectations of a high quality, high purity nucleic acid extraction with no apparent consequences for downstream processing- is it valuable to continue to report these?

 The reporting follows DToL templating and provides clear methodological information, sample and analysis metadata, and public accession links are active. The assembly is of high quality, as assessed by a suite of metrics, and will be a valuable addition to the genome catalogue.

Are sufficient details of methods and materials provided to allow replication by others?

Yes

Is the rationale for creating the dataset(s) clearly described?

Yes

Are the datasets clearly presented in a useable and accessible format?

Yes

Are the protocols appropriate and is the work technically sound?

Yes

Reviewer Expertise:

Bioinformatics, Genomics, Evolution

I confirm that I have read this submission and believe that I have an appropriate level of expertise to confirm that it is of an acceptable scientific standard.

# Reviewer report: Reviewer response for version 1

**DOI:** 10.21956/wellcomeopenres.27403.r134564

**Published:** 2025-10-16

**Authors:** Erin Toffelmier

**Affiliations:** 1 Virginia Polytechnic Institute and State University Department of Fish and Wildlife Conservation, Blacksburg, Virginia, USA

This article describes a genome assembly for *Apamea lithoxylaea* as part of the Darwin Tree of Life Project. The methods and results are technically sound, and the assembly metrics and completeness reflect a high-quality reference genome. 

 Notes:

 I suggest re-wording or clarify the sentence about the COI species identification method to reflect more directly that the method used followed Crowley et al., 2023. As it's written and cited, it suggests that this particular identification was carried out in Crowley et al., 2023. 

 The Hi-C methods sections are broken up with two different bolded subheadings while the HiFi section is not. It might be cleaner to have just one section for each method. 

 I think it would be good to state the SPRI ratios used throughout the HiC methods section. 

 A synteny analysis to support the naming of the Z and W chromosomes, along with any karyotype or other *a priori *expectation of genome size would make this more robust. 

Are sufficient details of methods and materials provided to allow replication by others?

Partly

Is the rationale for creating the dataset(s) clearly described?

Yes

Are the datasets clearly presented in a useable and accessible format?

Yes

Are the protocols appropriate and is the work technically sound?

Yes

Reviewer Expertise:

population and conservation genomics, ecology

I confirm that I have read this submission and believe that I have an appropriate level of expertise to confirm that it is of an acceptable scientific standard.
